# Body Positivity, Physical Health, and Emotional Well-Being Discourse on Social Media: Content Analysis of Lizzo’s Instagram

**DOI:** 10.2196/60541

**Published:** 2024-11-04

**Authors:** Stephanie L Albert, Rachel E Massar, Omni Cassidy, Kayla Fennelly, Melanie Jay, Philip M Massey, Marie A Bragg

**Affiliations:** 1 Department of Population Health NYU Grossman School of Medicine New York, NY United States; 2 Department of Medicine Department of Population Health NYU Grossman School of Medicine New York, NY United States; 3 Veterans Affairs New York Harbor, NY United States; 4 Department of Community Health Sciences Fielding School of Public Health University of California, Los Angeles Los Angeles, CA United States

**Keywords:** weight stigma, body positivity, health at every size, emotional well-being, social media, qualitative content analysis, well-being, social media, influencers, mental health outcomes, psychological health, body shaming, bullying

## Abstract

**Background:**

Weight stigma is a fundamental cause of health inequality. Body positivity may be a counterbalance to weight stigma. Social media is replete with weight-stigmatizing content and is a driver of poor mental health outcomes; however, there remains a gap in understanding its potential to mitigate the prevalence and impact of harmful messaging and to promote positive effects on a large scale.

**Objective:**

We selected musical artist Lizzo, whose brand emphasizes body positivity and empowerment, for an instrumental case study on the discourse on social media and specifically Instagram. We focused on 3 domains, including body positivity, physical health, and emotional well-being. These domains challenge social norms around weight and body size and have the potential to positively affect the physical and psychological health of people with diverse body sizes.

**Methods:**

We evaluated posts by Lizzo, comments from Instagram users, and replies to comments over a 2-month period (October 11 to December 12, 2019). Two coders rated Lizzo’s posts and Instagram users’ comments for their sentiments on the 3 domains. Replies to Instagram users’ comments were assessed for their reactions to comments (ie, did they oppose or argue against the comment or did they support or bolster the comment). Engagement metrics, including the number of “likes,” were also collected.

**Results:**

The final sample included 50 original posts by Lizzo, 250 comments from Instagram users, and 1099 replies to comments. A proportion of Lizzo’s content included body positive sentiments (34%) and emotional well-being (18%); no posts dealt explicitly with physical health. A substantial amount Instagram users’ comments and replies contained stigmatizing content including the use of nauseated and vomiting emojis, implications that Lizzo’s body was shameful and should be hidden away, accusations that she was promoting obesity, and impeachments of Lizzo’s health. In spite of the stigmatizing content, we also discovered content highlighting the beneficial nature of having positive representation of a Black woman living in a larger body who is thriving. Moreover, analysis of the discourse between users illustrated that stigmatizing expressions are being combated online, at least to some degree.

**Conclusions:**

This study demonstrates that Lizzo has exposed millions of social media users to messages about body positivity and provided more visibility for conversations about weight and shape. Future research should examine the extent to which body positive messages can lead to greater acceptance of individuals living in larger bodies. Instagram and other social media platforms should consider ways to reduce body-shaming content while finding ways to promote content that features diverse bodies. Shifting the landscape of social media could decrease stereotypes about weight and shape while increasing dialog about the need for greater acceptance and inclusion of people with diverse bodies.

## Introduction

Stigma is a fundamental cause of health inequality [1]. Weight stigma—“the social rejection and devaluation that accrues to those who do not comply with prevailing social norms of adequate body weight and shape” [2]—has received relatively less attention than other types of stigma. Weight stigma can be experienced through exposure to pervasive and deleterious stereotypes about individuals living in larger bodies, teasing or name calling, physical aggression, and social rejection [3]. Such experiences often cause individuals living in larger bodies to internalize stigma by adopting these societal beliefs about themselves and others [4]. For those in larger bodies, weight stigma is associated with depression [5,6], anxiety [7], substance use [5], low self-esteem [5,6], poor body image [6,8-10], increased social isolation [7-9], suicide risk [9-11], adverse academic outcomes [12,13], disordered eating [7,9,13-16], reduced physical activity [9,13,17,18], and increased overweight or obesity [16,19]. Body positivity may be a counterbalance to socially accepted weight stigma. Body positivity is “the philosophy that all people deserve to view themselves and their bodies in a positive light, regardless of how society dictates what is (and is not) the ‘ideal’ body type or beauty standard” [20]. Burgeoning research has demonstrated the benefits of being exposed to social media body positive content on individual-level internalized outcomes (eg, body satisfaction or dissatisfaction, body appreciation, mood, and self-esteem) [21-31]. Although body positivity has primarily been centered on internalized views of self, body positivity can be applied more broadly to having positive, inclusive, and transformative attitudes toward others with diverse bodies and shapes.

When people express weight stigmatizing views toward people in larger bodies, it is often rationalized as stemming from concern for health due to the presumption that body size is an accurate indicator of health. There is a widely accepted societal belief that people living in larger bodies cannot be physically or emotionally healthy. In contrast, the Health At Every Size (HAES) model espouses a holistic definition of health and rejects the assumption that people in larger bodies are unhealthy by default. The HAES model focuses on size acceptance, intuitive eating, and pleasurable movement—rather than on weight loss—to improve health [32]. Another aspect of the HAES model is that it recognizes that health and well-being is multidimensional, encompassing not only physical health but also social, spiritual, occupational, intellectual, and emotional well-being. While one’s emotional well-being is vulnerable to internalized weight stigma [33], the HAES model and body positivity may help to counteract the negative impacts of weight stigma and promote positive emotional well-being among people in larger bodies. Emerging evidence demonstrates that health interventions developed using HAES principles significantly improve participants’ depression, self-esteem, and body image avoidance behavior at follow-up compared to traditional diet interventions [34].

We live in a media-saturated environment that is replete with weight stigmatizing content. Instagram [35], for example, is one of the most popular social media platforms in the United States, with 133.5 million monthly users [36]. Almost half (47%) of US adults use Instagram, and it is particularly common among adults under 30 years, with 78% reporting they are Instagram users [37]. Similarly, almost three-quarters (72%) of US teens use Instagram [38]. A recent study by Instagram’s parent company suggests youth believe that Instagram contributes to poor mental health (eg, anxiety and depression) and less body satisfaction, particularly among female adolescent users [39]. Further, the US Surgeon General has issued an advisory identifying social media as a leading cause of the mental health crisis among youth [40]. Despite these concerns, social media continues to be a powerful source of health information [41], and there remains a gap in understanding its potential for mitigating the prevalence and impact of harmful messaging and for promoting positive effects on a large scale.

One internationally known musical artist that has been a central part of the discussion on weight and body size is Lizzo. She is a multi-Grammy award-winning singer and songwriter who has dominated pop, R&B, and rap charts since 2019 [42]. Her brand, “Black girl liberation” [43], highlights her intersectional social positions as a fat Black woman while acknowledging these positions are associated with oppression and inequality [44]. Her song lyrics emphasize body positivity and empowerment and she is widely recognized for sharing with fans and audiences the importance of appreciating one’s body, self-confidence as a Black and fat woman, and their importance to her and others’ mental health [45]. Lizzo shares these messages through easily accessible platforms, including Instagram, where she has 12.3 million followers. Given her massive platform and influence, her messaging around these topics provides an opposing narrative to deeply entrenched societal beliefs about weight and body size. Media effects processes suggest that repeated and continuous exposure to these types of messages will result in robust attitudinal, cognitive, emotional, and behavioral changes [46]. Thus, her messages have the potential to help those in larger bodies transform their own identities, while also driving change in attitudes, beliefs, and behaviors toward those in larger bodies at a population level.

We examined Lizzo’s Instagram posts as an instrumental case study of the social media discourse across 3 domains related to being in a larger body—body positivity, physical health in line with HAES, and emotional well-being. This design allowed us to study 1 case in depth to gain insight into these broader concepts across social media [47]. These three domains were selected as they challenge social norms around weight and body size and have the potential to positively affect the physical and psychological health of people with diverse body sizes. To assess the social media discourse, we captured 3 types of “nested” content (ie, original content (posts), comments to posts, and then replies to comments that are in response to, and grouped together, under original posts similar to a funnel). In the context of this study, we first assessed original content (posts) by the creator (Lizzo). Second, we assessed comments that Instagram users posted to Lizzo’s content. Third, we assessed posted replies to those comments. The primary goal of evaluating three complementary levels of content was to explore the degree to which more inclusive attitudes and behaviors exist in the face of malignant and ever-present weight-based stigma on social media. We hypothesized that Lizzo’s content would promote body positivity, contain references about her physical health that would reinforce HAES, and would include positive sentiments about her emotional well-being. We further hypothesized that there would be a disproportionate amount of negative/stigmatizing commentary on these domains from Instagram users. Lastly, we hypothesized that content promoting stigmatizing beliefs and attitudes would be combatted within the discourse between Instagram users.

## Methods

### Ethical Practices for Working With Social Media Data

This study was determined to be nonhuman subjects research per 45 CFR 46.102 (e)(1) [48]. Instagram users have the right to decide if they want their activity to be private and can do so by making their accounts private or limiting who can see their content. As such, this study only included posts, comments, and replies from public profiles that were part of the public domain. While the posts, comments, and replies were public, we took every precaution to deidentify data and protect rights to privacy consistent with recommendations [49]. This included (1) not collecting any data about personal accounts beyond what was in the screen grab images, (2) assigning IDs to comments and replies, (3) deidentifying data that were extracted and coded, and (4) paraphrasing extracted data for reporting or dissemination purposes. We also instructed our study team to not look at or follow Instagram users’ accounts or interact with their content.

### Selection of Posts, Comments, and Replies

While reviewing Lizzo’s Instagram account, the research team identified and screen captured Instagram posts during a 2-month time period (October 11 to December 12, 2019). The team determined a priori that a 2-month period of time would be feasible for manual data collection and would provide a reasonably comprehensive selection of Lizzo’s content as she frequently posted to her Instagram account. All posts were included in the sample that met the inclusion criteria of containing an image or video of Lizzo. Only one post during the 2-month data collection period was excluded because it did not prominently feature Lizzo. For each post, one member of the research team documented engagement metrics, including the number of “likes,” comments, and video views. Next, the team used a stratified purposeful sampling strategy for the selection of comments and replies. This included identifying 5 comments to each of Lizzo’s Instagram posts with the greatest amount of user engagement. User engagement was defined by the number of replies the comment received. If multiple comments for the same post had the same number of replies, the research team randomly selected 1 comment to stay in the sample. It also included the research team screen capturing the first 5 replies to each of the comments in the sample. Due to the low number of replies comments receive, with most receiving none, we expected that 5 replies would represent the overwhelming majority of all replies. When comments had fewer than 5 replies, all available replies were included.

### Codebook and Coding Procedures

The research team created a coding protocol to guide the content analysis of Instagram posts, Instagram user comments, and replies. To inform the development of our codebook, the researchers reviewed previous social media content analyses on body image and body positive themes [50-56]. The research team divided the codebook into three categories: (1) Lizzo Instagram posts, (2) Instagram users’ comments, and (3) replies to Instagram users’ comments. Within each category, the research team included comprehensive instructions and examples of when to apply codes in the codebook. The researchers coded Lizzo’s posts on the following domains: image type; how much of Lizzo’s body was shown in the post; what parts of Lizzo, if any, were exposed in the image; extent to which Lizzo’s clothing was revealing; assessment of whether the post was sexually suggestive; whether the post was promoting a product or a brand; sentiment about body positivity; sentiment about physical health; and sentiment about emotional well-being (see Table 1). The researchers coded Instagram users’ comments on the following domains: sentiment about body positivity, sentiment about physical health, and sentiment about emotional well-being. Lastly, the researchers assessed replies to Instagram users’ comments for their reaction to the comment (ie, did they oppose or argue against the comment or did they support or bolster the comment?).

**Table 1:** Codebook domains, description, and response options.

Variable		Description	Response options
**Lizzo posts**			
	Image type	What is included in the post	Picture only Picture and text on picture Video only Video and text on image
	Body depiction	How much of Lizzo’s body is shown in the post	Full body Partial body Face mostly Body fragmentation (ie, headless image in which a specific body part is featured) Multiple images shown
	Body exposure	Bare skin exposed for each of the following: (1) arms, (2) thigh(s)/leg(s), (3) midriff, (4) backside (butt), and (5) cleavage visible	Yes No Multiple images shown
	Clothing exposure	Extent to which Lizzo’s clothing is revealing in the post	Not at all revealing Moderately revealing Very revealing Not shown (ie, face only) Multiple images shown
	Sexual suggestiveness	Post is sexually suggestive (image or caption)	Yes No
	Promotion	Post is connected to or promotes an event or brand though mentions or tags	Yes No
	Body positivity	Sentiment toward body size in post (image and caption)	Negative (ie, fat shaming/weight stigmatization) Unrelated Positive (ie, body positivity/fat acceptance/self-love/all bodies are beautiful/body neutrality)
	Physical Health	Sentiment about physical health in post (image and caption)	Negative (ie, concern about health of those in larger bodies) Unrelated Positive (ie, supports physical health regardless of body size)
	Emotional well-being	Sentiment about emotional well-being in post	Negative (ie, negative well-being outcomes such as feeling sad, depressed, and lonely) Unrelated Positive (ie, positive well-being outcomes such as confidence, self-worth, empowerment, self-esteem)
**Instagram users’ comments**			
	Body positivity	How the comment refers to Lizzo’s body	Negative (ie, fat shaming/weight stigmatization) Unrelated Positive (ie, body positivity/fat acceptance/self-love/all bodies are beautiful/body neutrality)
	Physical health	Sentiment about physical health or medical conditions	Negative (ie, concern about health of those in larger bodies) Unrelated Positive (ie, supports health regardless of body size)
	Emotional well-being	Sentiment about emotional well-being outcomes	Negative (ie, negative well-being outcomes such as feeling sad, depressed, lonely) Unrelated Positive (ie, positive well-being outcomes such as confidence, self-worth, empowerment, self-esteem)
**Replies to Instagram users’ comments**			
	Reaction to comment	Whether the reply opposes/argues against or supports/bolters the comment	Opposes/argues against comment Unrelated Supports/bolsters comment No reply

There were 3 coders (SLA, REM, and KF)—all of whom identify as white women and one who lives in a larger body. The 3 coders met for a series of training sessions involving consensus coding and refinement of the codebook using approximately 20 of Lizzo’s posts that were not part of the sample. In addition to Lizzo’s original posts, Instagram users’ comments and replies to those comments were also coded and small modifications were made to the original coding scheme to improve clarity and increase consistency between coders. Following the training sessions, 1 round of pilot coding was conducted using the first 10 Lizzo Instagram posts, including the associated user comments and replies, in the sample. Posts were coded by 2 coders independently. At this stage, agreement was assessed using Krippendorff α reliability estimates. α Values ranged from as low as 0.24 to 1; the majority reached a satisfactory threshold of 0.67 [57]. Three variables (bare arms as part of body exposure, sexual suggestiveness, and body positivity) had low agreement between coders (0.24, 0.41, and 0.47, respectively). The research team met to discuss the pilot coding and resolve discrepancies from the first set of coded Instagram posts. This resulted in further refinement of the codebook. The remaining 40 posts were double coded by 2 coders in an effort to be conservative due to the range in reliability estimates. The coders merged their datasets once all posts, comments, and replies were assessed, discrepancies were identified, and any conflicts were resolved through group discussion with the coders and the first author. Coding agreement on individual variables ranged between 70% and 100% during the last stage of coding.

### Analyses

All analyses were conducted using SPSS (version 29; IBM Corp) [58]. Descriptive statistics (eg, frequencies, percentages, and ranges) were run for all variables in the study.

## Results

### Overview of Image Type and Attributes of Lizzo’s Posts

The final sample included 50 original posts by Lizzo, 250 comments from Instagram users (<1% of all comments), and 1099 replies to comments (17.9% of all replies). As of February 11, 2020, the 50 Instagram posts from Lizzo’s account that were in our sample received more than 26 million “likes,” and users left more than 300,000 comments (see Table 2). Posts with video clips (n=23) were viewed more than 51 million times. More than half (54%) of posts contained a picture only or a picture and text on the image, while the other 46% included video content. The largest proportion of Lizzo’s posts featured her full body (49%) followed by only her face (30.6%) and a smaller proportion included partial body (18.4%) or body fragmentation (ie, headless or specific body part featured; 2%). Body or skin exposure was common in Lizzo’s posts with bare thighs (46%), bare arms (32.7%), and cleavage (26.5%) displayed most often. Coders rated Lizzo’s attire as “moderately” to “very revealing” in almost half of the posts (47.6%), and more than a quarter of posts (28.0%) were rated as sexually suggestive. Finally, 46% of posts promoted an event or brand.

The primary aim of this paper was to document the social media discourse on body positivity and related constructs. The remainder of the findings are organized by these three themes: (1) body positivity, (2) physical health, and (3) emotional well-being. Within each theme, we first present a summary of Lizzo’s posts, then we describe the associated *positive* and *negative* Instagram users’ comments. Next, we characterize the *supporting* and *opposing* replies associated with the comments (see Table 3).

**Table 2:** Description of Lizzo’s posts (n=50)^a^.

		Values	Range	Total
Number of likes, mean		530,322.8	60,613-1,228,218	26,516,138
Number of comments, mean		6014.2	321-46,986	300,711
Number of views (video clips), mean		2,222,562.8	323,076-5,797,985	51,118,945
**Type of image**				
	Picture only	18 (36.0)	—^b^	—
	Picture and text on image	9 (18.0)	—	—
	Video only	15 (30.0)	—	—
	Video and text	8 (16.0)	—	—
**Body depiction**				
	Full body	24 (49.0)	—	—
	Partial body	9 (18.4)	—	—
	Face mostly	15 (30.6)	—	—
	Body fragmentation (headless/specific body part featured)	1 (2.0)	—	—
**Body exposure^c^**				
	Bare arms	16 (32.7)	—	—
	Bare thighs	23 (46.9)	—	—
	Bare midriff	6 (12.2)	—	—
	Bare gluteal (butt)	4 (8.2)	—	—
	Cleavage	13 (26.5)	—	—
**Clothing^d^**				
	Not at all revealing	22 (52.4)	—	—
	Moderately revealing	11 (26.2)	—	—
	Very revealing	9 (21.4)	—	—
	Sexually suggestive (yes)	14 (28.0)	—	—
	Branded content/promotional content (yes)	23 (46.0)	—	—

^a^All categories were mutually exclusive.

^b^Not applicable.

^c^The assessment of Lizzo’s body exposure was done using 49 posts as 1 post had multiple images.

^d^The assessment of Lizzo’s clothing was done using 42 posts because her clothing was not shown in 7 posts, and 1 post contained multiple images.

**Table 3:** Discourse and engagement on body positivity, health at every size, and emotional well-being domains associated with Lizzo’s Instagram posts.^a^

Parameter				Value
**Body positivity**				
	Lizzo’s posts, n			50
	Expressions of body positivity, n (%)			17 (34)
	**Comments on Lizzo’s posts (n=250)**			
		Positive comments, n (%)	7 (2.8)	
		Negative comments, n (%)	28 (11.2)	
		Likes for positive comments, n	565	
		Likes for negative comments, n	10,171	
	**Replies to positive comments (n=35)^b^**			
		Reply supports positive comment, n (%)	14 (40)	
		Reply opposes positive comment, n (%)	15 (42.8)	
		Likes for supportive replies, n	67	
		Likes for opposing replies, n	139	
	**Replies to negative comments (n=140)^b^**			
		Reply supports negative comment, n (%)	51 (36.4)	
		Reply opposes negative comment, n (%)	56 (40)	
		Likes for supportive replies, n	694	
		Likes for opposing replies, n	1223	
**Physical health**				
	Lizzo’s posts, n			50
	Posts supporting physical health, n (%)			0 (0)
	**Comments on Lizzo’s posts (n=250)**			
		Positive comments, n (%)	0 (0)	
		Negative comments, n (%)	6 (2.4)	
		Likes for positive comments, n	0	
		Likes for negative comments, n	2771	
	**Replies to positive comments (n=0)^b^**			
		Reply supports positive comment, n (%)	0 (0)	
		Reply opposes positive comment, n (%)	0 (0)	
		Likes for supportive replies, n	0	
		Likes for opposing replies, n	0	
	**Replies to negative comments (n=30)^b^**			
		Reply supports negative comment, n (%)	9 (30)	
		Reply opposes negative comment, n (%)	19 (63.3)	
		Likes for supportive replies, n	137	
		Likes for opposing replies, n	333	
**Emotional well-being**				
	Lizzo’s posts, n			50
	Supports emotional well-being, n (%)			9 (18)
	**Comments on Lizzo’s posts (n=250)**			
		Positive comments, n (%)	11 (4.4)	
		Negative comments, n (%)	6 (2.4)	
		Likes for positive comments, n	2010	
		Likes for negative comments, n	3254	
	**Replies to positive comments (n=55)**			
		Reply supports positive comment, n (%)	16 (29.1)	
		Reply opposes positive comment, n (%)	10 (18.2)	
		Likes for supportive replies, n	80	
		Likes for opposing replies, n	304	
	**Replies to negative comments (n=30)**			
		Reply supports negative comment, n (%)	16 (53.3)	
		Reply opposes negative comment, n (%)	11 (36.7)	
		Likes for supportive replies, n	310	
		Likes for opposing replies, n	260	

^a^Because comments and replies could be coded as “unrelated” percentages may not total 100%.

^b^We assessed the first 5 replies to each positive and negative comment; therefore, the number of replies shown depends on the comments. Across all 250 comments, we assessed 1099 replies.

### Body Positivity (vs Weight Stigma)

**Figure 1:**
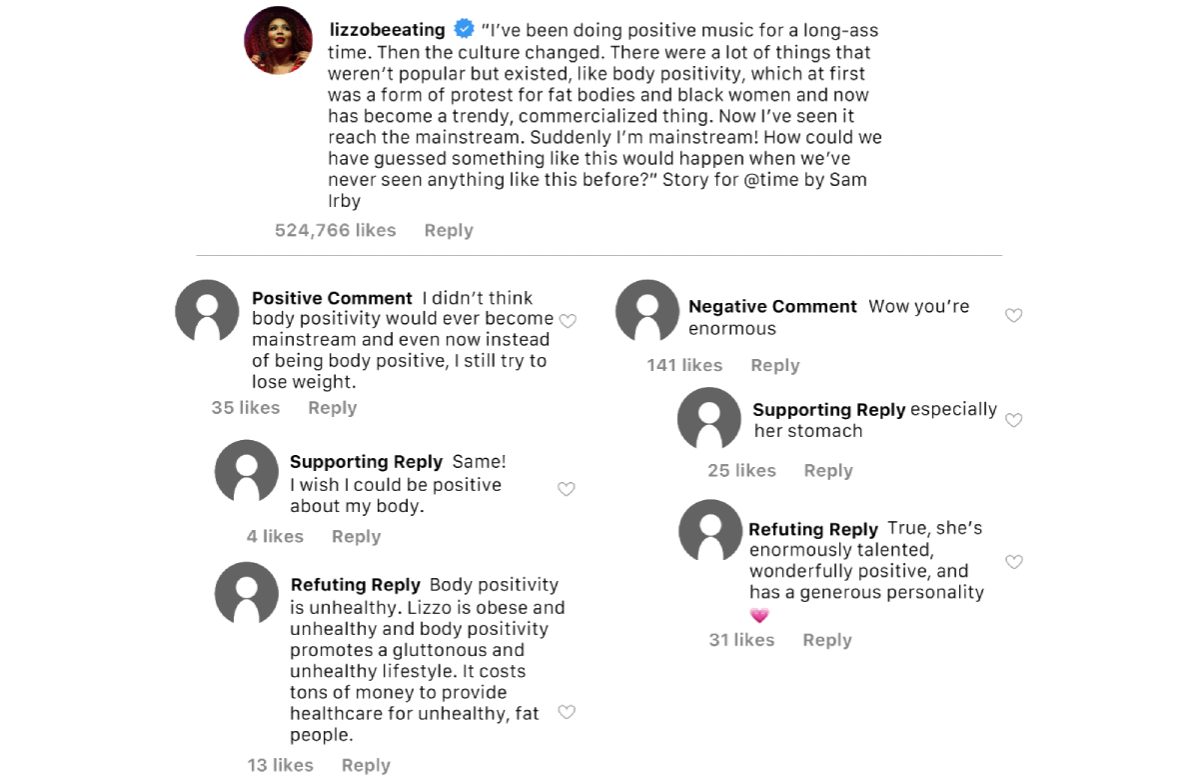
Sample body positivity discourse.

Lizzo’s Instagram posts, Instagram users’ comments, and replies to users’ comments were coded as promoting body positivity if the image, video, caption, or comment were perceived to have included body positive, fat acceptance, fat liberation, body neutrality, all bodies are beautiful, or other body inclusive sentiments (See Figure 1 for sample discourse related to body positivity from one of Lizzo’s posts). Body positivity was the most frequent theme to appear across all of Lizzo’s Instagram posts as it was found to be present in more than a third (n=17, 34%) of posts. This included references to loving one’s body, being fat and beautiful, and unapologetically and confidently showing one’s body.

Across the 250 Instagram users’ comments sampled, only 7 (2.8%) were positively related to body positivity and those positive comments earned 565 “likes.” Such comments included praising Lizzo for her body positive content, clapping and cheering emojis, and sentiments echoing body positive ideals. Four times as many comments included negative or stigmatizing language related to body positivity (n=28, 11.2%). Negative comments also earned 10,171 “likes,” or 18 times the number of “likes” than the positive comments received. Examples of negative comments included fat-shaming language, name-calling, calls for Lizzo to cover up, and emojis depicting disgust such as vomiting.

In total, 35 replies to positive comments in support of body positivity were assessed. Less than one-half (n=14) of the replies were supportive of the positive comment, while a similar number (n=15) opposed the positive comment (ie, included stigmatizing sentiment). The replies supporting positive comments earned 67 “likes” whereas the replies opposing positive comments (ie, they contained stigmatizing language) received 139 “likes.”

In total, 140 replies to negative or body stigmatizing comments were assessed. Roughly equal numbers of those replies were supportive of the negative comments (n=51) and in opposition to the negative comments (ie, were body positive; n=56). Almost twice as many people “liked” the replies opposing the negative or body stigmatizing comments as compared to the replies that were supporting (ie, stigmatizing) the negative comments (1223 vs 694).

### Physical Health

**Figure 2:**
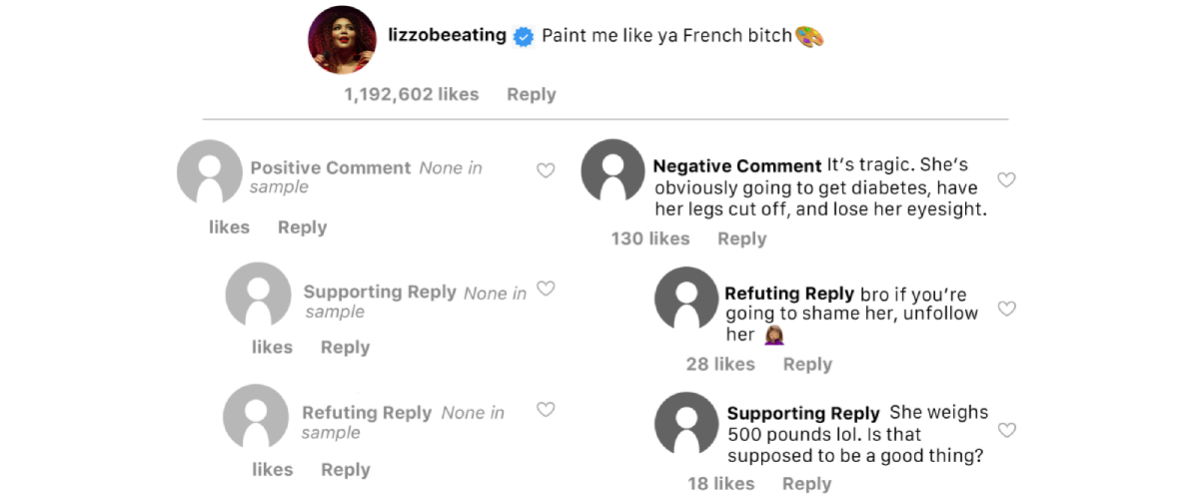
Sample physical health discourse.

Lizzo’s Instagram posts, Instagram users’ comments, and replies to users’ comments were coded as promoting physical health in line with HAES if the image, video, caption, or comment were perceived to have sentiments that support physical health regardless of body size (see Figure 2 for sample discourse related to physical health from one of Lizzo’s posts). While none of Lizzo’s posts explicitly promoted physical health, 6 (2.4%) comments were left on Lizzo’s posts related to her perceived poor health or the poor health of people living in larger bodies. Such comments included references to disease progression, assertions that being fat is unhealthy, and therefore, should not be promoted, health is the result of individual-level behaviors, and the high cost of treating weight-related illness. Those negative comments received 2771 “likes.” Thirty replies to negative comments were assessed. Nine of those replies (30%) supported the negative comments that were antithetical to physical health in line with HAES and those replies earned 137 “likes.” Conversely, more than 2 times as many replies (n=19) opposed the comments (ie, included sentiments that support physical health in line with HAES) and those replies earned 333 “likes.”

### Emotional Well-Being

**Figure 3:**
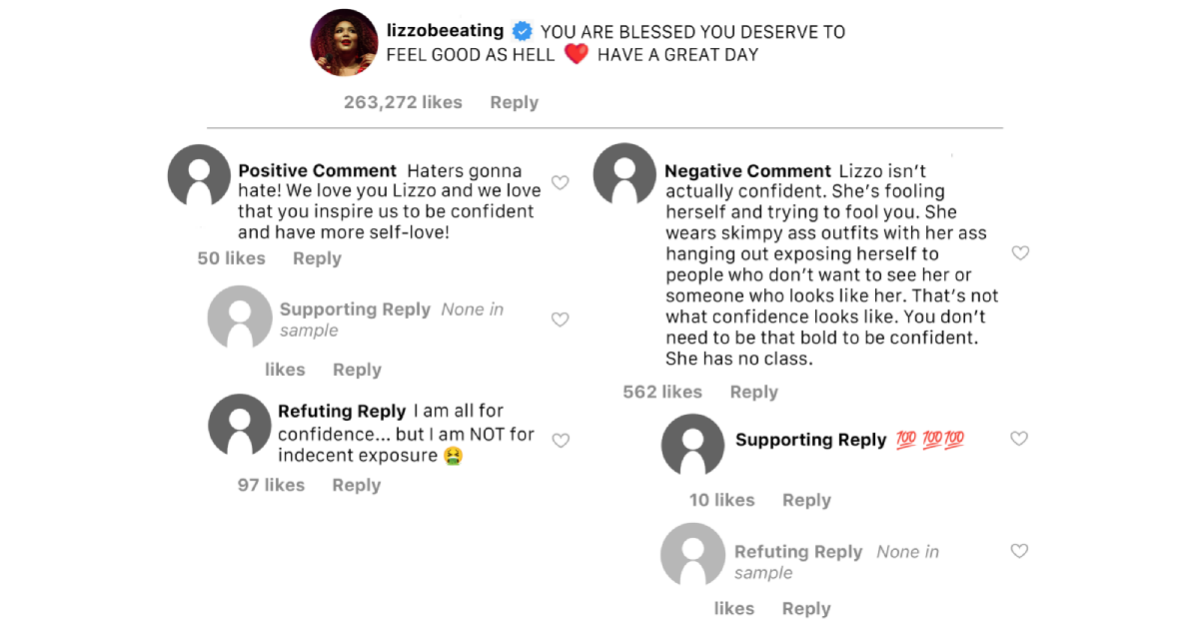
Sample emotional well-being discourse.

Lizzo’s Instagram posts, Instagram users’ comments, and replies to users’ comments were coded as promoting emotional well-being if the image, video, caption, or comment were perceived to have sentiments that indicate positive mental health outcomes (eg, self-esteem, confidence, and happiness) or behaviors that are associated with emotional well-being (eg, relaxation techniques, self-care, support, or social connections) [59] (see Figure 3 for sample discourse related to emotional well-being from one of Lizzo’s posts). Content related to promoting emotional well-being was observed in 18% (n=9) of Lizzo’s posts. Specific content included references to self-love, self-acceptance, independence, confidence, empowerment, feeling “good as hell,” and holding oneself to your own standards rather than making comparisons to others.

Across the 250 Instagram users’ comments assessed, 11 (4.4%) were positive comments related to emotional well-being and those positive comments earned 2010 “likes.” Positive comments included statements about how Lizzo has inspired them, helped them with their confidence, served as a role model for how to love oneself, and praised Lizzo for her messaging about positive emotional well-being. About half as many comments included negative or opposing viewpoints as they relate to the emotional well-being of Lizzo or someone in a larger body (n=6, 2.4%). Examples of negative comments included critiques of the way in which Lizzo expressed confidence and self-love, claims that her expression of confidence was inappropriate and feigned, and an overall inability to believe that Lizzo or others living in a larger body could thrive emotionally. Despite being fewer in numbers, negative comments earned more “likes” (n=3254) than positive comments.

The sample included 55 replies to positive comments related to emotional well-being. More than a quarter (n=16) of the replies were supportive of the positive comment and these replies earned 80 “likes.” Conversely, 10 replies were coded as opposing the positive comment (ie, included language or sentiment related to adverse mental health behaviors and outcomes) and those replies earned 304 “likes.” That was nearly 4 times as many “likes” as replies supportive of positive comments received.

Thirty replies were coded in response to negative well-being comments. More than half (n=16) of the replies supported the negative comment and received 310 “likes,” while a little more than a third (n=11) opposed the negative comment and earned 260 “likes.”

## Discussion

Our team conducted an instrumental case study, using a content analysis, to characterize content on Instagram related to body positivity, physical health, and emotional well-being. We analyzed Lizzo’s personal Instagram content and other Instagram users’ reactions, as well as discourse between users related to her content. As expected, we found that Lizzo’s content included body positive sentiments. Similarly, we found content related to emotional well-being. No posts in our sample dealt explicitly with physical health, though this is something that Lizzo has publicly discussed [60].

Our analyses revealed a substantial amount of negative commentary among Instagram users on Lizzo’s content that stigmatized people in larger bodies in the 3 domains we examined. Regardless of what was posted by Lizzo, comments and replies included fatphobic and harmful stigmatizing content. People used nauseated and vomiting emojis, implied her larger body was shameful and should be hidden away or covered up, and said she was promoting obesity. Similarly, commentors stated that one cannot be healthy and fat and that health care costs are only appropriate for people that are not fat (ie, for those whose health outcomes are not a result of perceived personal behaviors). Expressly related to body positivity and physical health, negative comments left by Instagram users outnumbered the positive comments several fold. These findings are consistent with past studies that have documented how rampant weight stigma is on social media in addition to misinformation circulating about the etiology of obesity [61]. This also mirrors individual experiences with weight stigma where 25%-50% all youth report being bullied due to their weight [62].

In spite of the potentially harmful content, we also discovered content that promotes greater acceptance of those in larger bodies. Instagram users shared how beneficial it was to see someone that looks like them who is confident in their body and who appears to be thriving. And although more negative Instagram users’ comments were found related body positivity and physical health, there were greater numbers of replies that opposed negative sentiments and that opposition earned more “likes.” This suggests that commentary promoting stigmatizing beliefs and attitudes are being combated online, at least to some degree. Findings related to emotional well-being were more complicated and mixed and may be due to the impact of weight stigma on mental health for those living in larger bodies and the generally accepted belief that one must be struggling emotionally if they do not conform to socially accepted body size.

Past research shows that social media influencers and celebrities are incredibly powerful actors for influencing attitudes and behavioral intentions. In one lab study, for example, preadolescent boys preferred food products endorsed by sports celebrities more than food products endorsed by non-celebrities [63]. Another lab study among 9 to 11-year-old children found that children who were exposed to food endorsed by influencers consumed more calories than children who saw influencers endorsing nonfood products. This effect is particularly relevant in the context of music celebrities, because children and adolescents report spending 2.5 hours listening to music each day [64], and Black and Latinx youth report spending 3 hours listening to music daily [65]. Furthermore, adolescents are sensitive to social cues that indicate popularity (eg, number of “followers,” “likes,” and views), [66] which makes them more susceptible to changes in ideals and beliefs based on content from celebrities who are active on social media [67]. With 12.3 million followers and more than 3000 posts [68], Lizzo likely recognizes the impact that social media engagement has had on her career and continues to become a potent, and active, social media spokesperson for advancing discourse on body positivity.

As a fat Black woman, Lizzo’s messaging—and the responses to her messaging—highlights the intersectionality of race or ethnicity, weight status, and gender identity and the associated oppression each of these social positions carry. Representation within each of these categories compound in ways that are challenging, but necessary, to recognize and uphold. The intersectionality of these social positions is key to understanding individuals’ experiences and identify levers of change and support. Black and Latinx youth in larger bodies, for instance, experience intersectional discrimination, which may uniquely affect mental health [69]. Black and Latinx communities tend to be more accepting of larger people and are perceived to be protected from weight stigma and its negative effects [20]. Weight stigma in these communities persists, however, likely due to the intersectional nature that violates racial, body size, and gender norms simultaneously [70]. There is a dearth of research regarding this intersectionality and its effect on mental health and behaviors.

### Limitations

There are a few limitations to this study. First, we examined a sample of posts, users’ comments, and replies to users’ comments on the original content of one musical artist on one social media platform, which may not fully capture the body positivity, physical health, or emotional well-being message landscapes on social media. Analyzing data like those within this analysis provides critical information to support our understanding of the body positivity movement and its potential to improve population health outcomes. The second limitation of this work is that comments and images can be removed by social media platform creators for allegedly violating standards, which may limit the representativeness of our sample. Although we are unable to quantify whether anything was removed, the primary objective was to focus on overarching themes rather than specific occurrences. Third, we do not have any information on the Instagram users who left comments or replies, regardless of whether they were positive or negative. This may introduce bias into this study as those who actively engage with social media content may differ in important ways from those that are more passive consumers. Fourth, it was outside of the scope of this study to explicitly measure the influence of Lizzo’s posts on individuals’ attitudes or behaviors toward those in larger bodies. It will be important for future studies to identify the associations with body positive messages and individual outcomes. Last, these data were collected during a time when body positivity was gaining momentum [71] albeit in a more limited internalized way. The pendulum may be shifting back toward less inclusive attitudes with a renewed focus on weight loss [72]. Somewhat related is the fact that some believe that the HAES movement discourages the use of treatments such as bariatric surgery and anti-obesity medications, potentially stigmatizing individuals who seek these treatments to improve their health [73,74]. Thus, our findings may not reflect the current social media discourse on body positivity and related domains (ie, physical health and emotional well-being).

### Conclusions

This study demonstrates that Lizzo has created opportunities for millions of social media users to be exposed to messages about body positivity, which has generated dialog about weight-based discrimination and bias and provides more visibility for conversations about weight and shape. Future research should examine the extent to which body positive images can lead to greater acceptance of individuals living in larger bodies. Studies could examine the ability of body positivity—promoted by popular celebrities and influencers—to decrease weight-based discrimination and bias among society more broadly. The findings also indicate that some social media users respond to body positivity content with weight-stigmatizing comments on posts. Given that stigmatizing language is hurtful, persists on social media, and leads to poor mental and physical health outcomes, Instagram and other social media platforms should consider ways to reduce body-shaming content while finding ways to promote or amplify content that features acceptance of diverse bodies. Shifting the landscape of social media in these ways would create opportunities to decrease stereotypes about weight and shape while increasing dialog about the need for greater acceptance and inclusion of people with diverse bodies.

## Abbreviations

HAES: Health at every size

## References

[1] Hatzenbuehler ML, Phelan JC, Link BG (2013). Stigma as a fundamental cause of population health inequalities. Am J Public Health.

[2] Tomiyama AJ, Carr D, Granberg EM, Major B, Robinson E, Sutin AR, Brewis A (2018). How and why weight stigma drives the obesity 'epidemic' and harms health. BMC Med.

[3] Puhl R (2008). Weight Discrimination: A Socially Acceptable Injustice.

[4] Bennett BL, Wagner AF, Latner JD (2022). Body checking and body image avoidance as partial mediators of the relationship between internalized weight bias and body dissatisfaction. Int J Environ Res Public Health.

[5] Bucchianeri MM, Eisenberg ME, Wall MM, Piran N, Neumark-Sztainer D (2014). Multiple types of harassment: associations with emotional well-being and unhealthy behaviors in adolescents. J Adolesc Health.

[6] Eisenberg ME, Neumark-Sztainer D, Haines J, Wall M (2006). Weight-teasing and emotional well-being in adolescents: longitudinal findings from project EAT. J Adolesc Health.

[7] Goldfield G, Moore C, Henderson K, Buchholz A, Obeid N, Flament M (2010). The relation between weight-based teasing and psychological adjustment in adolescents. Paediatr Child Health.

[8] Hayden-Wade HA, Stein RI, Ghaderi A, Saelens BE, Zabinski MF, Wilfley DE (2005). Prevalence, characteristics, and correlates of teasing experiences among overweight children vs. non-overweight peers. Obes Res.

[9] Puhl RM, Latner JD (2007). Stigma, obesity, and the health of the nation's children. Psychol Bull.

[10] Slater A, Tiggemann M (2011). Gender differences in adolescent sport participation, teasing, self-objectification and body image concerns. J Adolesc.

[11] Douglas VJ, Kwan MY, Gordon K (2021). The roles of weight stigma, emotion dysregulation, and eating pathology in suicide risk. Body Image.

[12] MacCann C, Roberts RD (2013). Just as smart but not as successful: obese students obtain lower school grades but equivalent test scores to nonobese students. Int J Obes (Lond).

[13] Puhl RM, Luedicke J (2012). Weight-based victimization among adolescents in the school setting: emotional reactions and coping behaviors. J Youth Adolesc.

[14] King KM, Puhl RM, Luedicke J, Peterson JL (2013). Eating behaviors, victimization, and desire for supportive intervention among adolescents in weight-loss camps. Eat Behav.

[15] Haines J, Neumark-Sztainer D, Eisenberg ME, Hannan PJ (2006). Weight teasing and disordered eating behaviors in adolescents: longitudinal findings from project EAT (eating among teens). Pediatrics.

[16] Haines J, Kleinman KP, Rifas-Shiman SL, Field AE, Austin SB (2010). Examination of shared risk and protective factors for overweight and disordered eating among adolescents. Arch Pediatr Adolesc Med.

[17] Losekam S, Goetzky B, Kraeling S, Rief W, Hilbert A (2010). Physical activity in normal-weight and overweight youth: associations with weight teasing and self-efficacy. Obes Facts.

[18] Jensen CD, Steele RG (2009). Body dissatisfaction, weight criticism, and self-reported physical activity in preadolescent children. J Pediatr Psychol.

[19] Quick V, Wall M, Larson N, Haines J, Neumark-Sztainer D (2013). Personal, behavioral and socio-environmental predictors of overweight incidence in young adults: 10-yr longitudinal findings. Int J Behav Nutr Phys Act.

[20] Fuhlendorf H (2021). DriveThru.

[21] Nelson SL, Harriger JA, Miller-Perrin C, Rouse SV (2022). The effects of body-positive Instagram posts on body image in adult women. Body Image.

[22] Cowles E, Guest E, Slater A (2023). Imagery versus captions: the effect of body positive Instagram content on young women's mood and body image. Body Image.

[23] Parcell L, Jeon S, Rodgers RF (2023). Effects of COVID-19 specific body positive and diet culture related social media content on body image and mood among young women. Body Image.

[24] Becker E, Rodgers RF, Zimmerman E (2022). #Body goals or #Bopo? Exposure to pregnancy and post-partum related social media images: effects on the body image and mood of women in the peri-pregnancy period. Body Image.

[25] Tiggemann M, Anderberg I, Brown Z (2020). #Loveyourbody: the effect of body positive Instagram captions on women's body image. Body Image.

[26] Manning TM, Mulgrew KE (2022). Broad conceptualisations of beauty do not moderate women's responses to body positive content on Instagram. Body Image.

[27] Dhadly PK, Kinnear A, Bodell LP (2023). #BoPo: Does viewing body positive TikTok content improve body satisfaction and mood?. Eat Behav.

[28] Davies B, Turner M, Udell J (2020). Add a comment … how fitspiration and body positive captions attached to social media images influence the mood and body esteem of young female Instagram users. Body Image.

[29] Fasoli F, Ogden J, Johnson S (2023). Body positivity or humorous parody? The impact of Instagram imagery on body image concerns. J Psychol.

[30] Di Michele D, Guizzo F, Canale N, Fasoli F, Carotta F, Pollini A, Cadinu M (2023). #SexyBodyPositive: when sexualization does not undermine young women's body image. Int J Environ Res Public Health.

[31] Fardouly J, Slater A, Parnell J, Diedrichs PC (2023). Can following body positive or appearance neutral Facebook pages improve young women's body image and mood? Testing novel social media micro-interventions. Body Image.

[32] Association for Size Diversity and Health (2020). Health At Every Size Principles.

[33] Puhl R, Suh Y (2015). Health consequences of weight stigma: implications for obesity prevention and treatment. Curr Obes Rep.

[34] Bacon L, Stern JS, Van Loan MD, Keim NL (2005). Size acceptance and intuitive eating improve health for obese, female chronic dieters. J Am Diet Assoc.

[35] META (2024). Instagram.

[36] OBERLO (2024). Instagram Users in the US (2019-2026).

[37] Pew Research Center (2024). Americans' Social Media Use.

[38] Pew Research Center (2021). Social Media Use in 2021.

[39] Wells G, Horwitz J, Setharaman D (2021). Facebook knows Instagram is toxic for teen girls, company documents show. The Wall Street Journal Internet.

[40] Office of the Surgeon General (2021). Publications and Reports of the Surgeon General. Protecting Youth Mental Health: The US Surgeon General's Advisory.

[41] Strasburger VC, Jordan AB, Donnerstein E (2010). Health effects of media on children and adolescents. Pediatrics.

[42] (2019). Lizzo named The Associated Press' Entertainer of the Year.

[43] Arensbak H (2017). Lizzo proudly calls herself a 'fat' woman. Are we allowed to as well?. The Conversation.

[44] Collins HP, Bilge S (2020). Intersectionality (Key Concepts).

[45] Carlin S (2019). Bustle.

[46] Bryant J, Zillmann D, Nabi ROM (2009). A Retrospective and Prospective Look at Media Effects.

[47] Crowe S, Cresswell K, Robertson A, Huby G, Avery A, Sheikh A (2011). The case study approach. BMC Med Res Methodol.

[48] (2018). 45 CFR Part 46.

[49] Moreno MA, Goniu N, Moreno PS, Diekema D (2013). Ethics of social media research: common concerns and practical considerations. Cyberpsychol Behav Soc Netw.

[50] Cohen R, Irwin L, Newton-John T, Slater A (2019). #bodypositivity: a content analysis of body positive accounts on Instagram. Body Image.

[51] Alberga AS, Withnell SJ, von Ranson KM (2018). Fitspiration and thinspiration: a comparison across three social networking sites. J Eat Disord.

[52] Boepple L, Thompson JK (2016). A content analytic comparison of fitspiration and thinspiration websites. Int J Eat Disord.

[53] Simpson CC, Mazzeo SE (2017). Skinny is not enough: a content analysis of fitspiration on pinterest. Health Commun.

[54] Webb JB, Vinoski ER, Bonar AS, Davies AE, Etzel L (2017). Fat is fashionable and fit: a comparative content analysis of fatspiration and health at every size Instagram images. Body Image.

[55] Ghaznavi J, Taylor LD (2015). Bones, body parts, and sex appeal: an analysis of #thinspiration images on popular social media. Body Image.

[56] Boepple L, Ata RN, Rum R, Thompson JK (2016). Strong is the new skinny: a content analysis of fitspiration websites. Body Image.

[57] Beckler DT, Thumser ZC, Schofield JS, Marasco PD (2018). Reliability in evaluator-based tests: using simulation-constructed models to determine contextually relevant agreement thresholds. BMC Med Res Methodol.

[58] IBM Corp (2022). IBM SPSS Statistics for Windows.

[59] Centers for Disease Control and Prevention (2023). Tips to Improve Your Emotional Well-Being.

[60] Etienne V (2023). Lizzo talks exercising, focusing on her health without "Trying to Escape Fatness". People.

[61] Westbury S, Oyebode O, van Rens T, Barber TM (2023). Obesity stigma: causes, consequences, and potential solutions. Curr Obes Rep.

[62] Puhl RM, Lessard LM (2020). Weight stigma in youth: prevalence, consequences, and considerations for clinical practice. Curr Obes Rep.

[63] Dixon H, Scully M, Niven P, Kelly B, Chapman K, Donovan R, Martin J, Baur LA, Crawford D, Wakefield M (2014). Effects of nutrient content claims, sports celebrity endorsements and premium offers on pre-adolescent children's food preferences: experimental research. Pediatr Obes.

[64] (2024). Listening to Music and Watching Music Videos.

[65] Rideout VJ, Foehr UG, Roberts DF (2010). Generation M2: media in the lives of 8-to 18-year-olds. The Kaiser Family Foundation.

[66] Sherman LE, Hernandez LM, Greenfield PM, Dapretto M (2018). What the brain 'Likes': neural correlates of providing feedback on social media. Soc Cogn Affect Neurosci.

[67] Lutfeali S, Ward T, Greene T, Arshonsky J, Seixas A, Dalton M, Bragg MA (2020). Understanding the extent of adolescents' willingness to engage with food and beverage companies' Instagram accounts: experimental survey study. JMIR Public Health Surveill.

[68] Lizzo (2024). @lizzobeeating Instagram.

[69] Smith CA (2019). Intersectionality and sizeism: implications for mental health practitioners. Women & Therapy.

[70] Strings S (2019). Fearing the Black Body: The Racial Origins of Fat Phobia.

[71] Gelsinger AS (2021). A critical analysis of the body positive movement on Instagram: how does it really impact body image?. Spectra Undergraduate Research Journal.

[72] Gaffney T (2023). There's a huge fatphobia problem in the eating disorder world?: Even in treatment, weight stigma fails patients. Stat.

[73] Terry PE (2023). New obesity guidance for pediatrics: medicalizing obesity or acquiescing to our obesogenic culture?. Am J Health Promot.

[74] Kyle T (2022). Health at Every Size or One Size Fits All?. ConscienHealth.

